# Multiple magnetic phase transitions with different universality classes in bilayer La$_{1.4}$Sr$_{1.6}$Mn$_{2}$O$_7$ manganite

**DOI:** 10.1038/s41598-021-00544-8

**Published:** 2021-10-27

**Authors:** Birendra Kumar, Jeetendra Kumar Tiwari, Harish Chandr Chauhan, Subhasis Ghosh

**Affiliations:** School of Physical Sciences, Jawaharlal Nehru University, New Delhi, 110067 India

**Keywords:** Materials science, Physics

## Abstract

Here, we report three magnetic transitions at 101 K (T$_{C1}$), 246 K (T$_{C2}$) and 295 K (T$_{C3}$) in bilayer La$_{1.4}$Sr$_{1.6}$Mn$_{2}$O$_7$. The second order phase transitions have been identified at these transition points with the help of change in entropy analysis and modified Arrott plots (MAPs). The critical behavior around T$_{C1}$, T$_{C2}$ and T$_{C3}$ have been studied by MAPs and Kouvel–Fisher method. Based on these analyses four magnetic phases are: (1) 2D Ising ferromagnetic (FM) below T$_{C1}$,(2) 2D Heisenberg canted antiferromagnetic (CAFM-I) and FM clusters in temperature range T$_{C1}$ < T < T$_{C2}$, (3) 2D Heisenberg CAFM-II and FM clusters with non magnetically interacting planes in temperature range T$_{C2}$ < T < T$_{C3}$ and (4) paramagnetic for T > T$_{C3}$.

## Introduction

**Figure 1:**
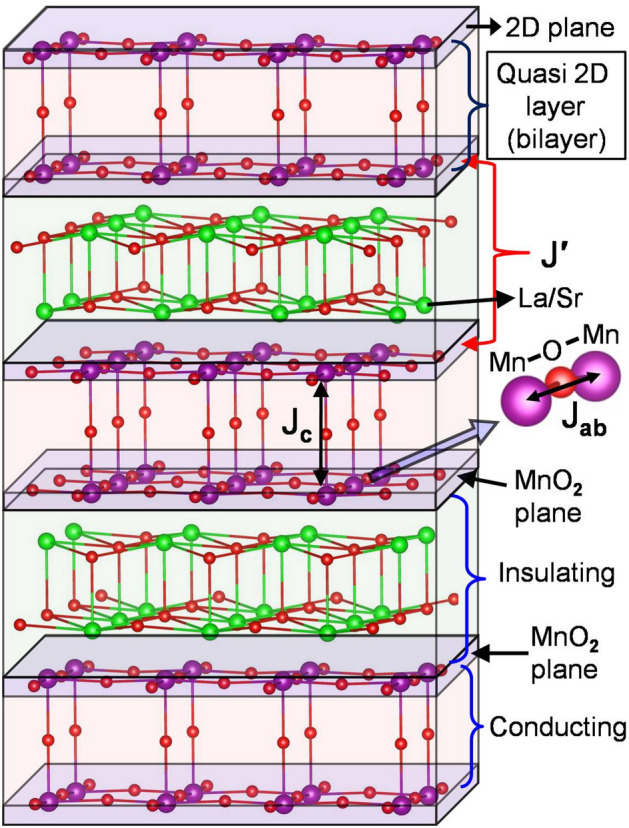
Schematic diagram of stacked bilayer La$_{2-2x}$Sr$_{1+2x}$Mn$_{2}$O$_{7}$: two consecutive bilayers are separated by insulating layer. The parameters J$'$, J$_{c}$ and J$_{ab}$ are magnetic interaction strength between Mn ions in MnO$_{2}$ planes separated by an insulating layer, Mn ions of different MnO$_{2}$ planes in the bilayer and Mn ions in a MnO$_{2}$ plane, respectively.

Two-dimensional (2D) materials, due to their versatile transport, optical^1^, thermal and mechanical properties, and their applications in various kind of devices^2,3^, become the subject of intense research activities. Though, magnetism in Van der Waals monolayer and hetrostructures is an active area of research^4,5^, but a conspicuous missing field of the research activities of conventional (non Van der Waals systems) 2D materials is their magnetic properties. Generally, theorists are pessimistic about spontaneous magnetism and magnetic phase transition in 2D systems. All the three-dimensional (3D) systems show magnetic phase transition at a finite temperature while in the one-dimension long-range ordering is possible only at absolute zero temperature^6,7^. But, the 2D systems being at the border of these two extremes, leads to a complex situation. In 2D system, the existence of long-range order at finite temperature strongly depends on spin dimensionality *n*, which is determined by the physical parameters of the systems^8^. According to the Hohenberg–Mermin–Wagner theorem^6,7^, thermal fluctuations destroy the long-range magnetic order in 2D systems at any finite temperature for spin dimension $n = 3$ because the continuous symmetry of isotropic Heisenberg model leads to gapless long wavelength excitations (spin waves)^9^. For spin dimensionality $n = 1$, the exact solution of 2D Ising model^10,11^ shows that a phase transition from disordered phase to magnetically ordered phase occurs at T$_{C}$ > 0. Ising model is the simplified version of the isotropic Heisenberg model in which nearest neighbor interactions are considered. In Ising model non-diagonal terms of the spin matrices are neglected, leading to stabilization of ferromagnetism in 2D. In this case, anisotropic exchange interaction can be given by Hamiltonian:

1 $$\begin{aligned} H = -\frac{1}{2}\sum _{ij}\left( J_{x}S_{i}^{x}S_{j}^{x} + J_{y}S_{i}^{y}S_{j}^{y} + J_{z}S_{i}^{z}S_{j}^{z}\right) \end{aligned}$$

where $J _{x}$, $J _{y}$, and $J _{z}$ are exchange strength in x, y, and z directions, respectively and they are unequal. In addition to anisotropic exchange interaction or Ising interaction, dipole-dipole interaction, external magnetic field and interaction between different layers in 2D system may also stabilize spontaneous magnetization^12^. In particular, dipole-dipole interaction will be important in systems with centrosymmetry, such as manganites. In case of non-centrosymmetric systems, another anisotropic exchange interaction called Dzyaloshinskii Moriya interaction can also help in stabilization of ferromagnetism/antiferromagnetism in 2D systems^13^. Here, the anisotropy of the system favors a specific spin component which opens a gap in the spin wave spectrum that suppress the effect of thermal fluctuations. The planar 2D magnets ($n = 2$), described by XY model, shows no transition from disordered state to long-range ordered state, although the susceptibility diverges below a finite temperature. Berezinskii^14^, Kosterlitz and Thouless^15^ have shown that this divergence is associated with an onset of topological order which is characterized by an algebraic decay of spin correlations and by the presence of bound pairs of vortex and anti-vortex arrangements of spins. Hence, below the Kosterlitz-Thouless temperature T$_{KT}$, quasi long-range magnetic order is established and the existence of a finite order parameter is suppressed only marginally with the system size. It is very difficult to achieve true 2D magnetic crystals^16–18^. The critical phenomena has been experimentally validated in thin films of magnetic materials^19,20^ or in 3D layered transition metal compounds^21^, which is a stack of weakly-coupled 2D magnetic layers. Most of the 2D magnetic systems either show 2D Ising ferromagnetic (FM)^22^ or 2D Heisenberg^23–25^ and 2D XY^26–28^ with antiferromagnetic (AFM) coupling between nearest neighbor spins or their crossover from one 2D phase to other 2D phase caused by reorientation of spins with temperature. In this context, it will be worthwhile to investigate in detail the anisotropic exchange interaction on the magnetic properties and magnetic phase transition in quasi-two-dimensional (Q2D) magnetic layers embedded in 3D matrix, such as bilayer manganites La$_{2-2x}$Sr$_{1+2x}$Mn$_{2}$O$_{7}$. Moreover, recently bilayer manganites have received reneard attention due to the observation of different topological spin structures, such as skyrmion bubbles and biskyrmion^29,30^. Hence, it is more relevant to investigate phase transition and critical phenomena in bilayer manganite to reveal the nature of the exchange interaction responsible for these topological spin structures. For the layered systems, Eq. (1) may be modified by substituting $J _{x}$ = $J _{y}$ = $J _{ab}$ and $J _{z} = (J' + J _{c}$), the parameters J$_{ab}$, J$_{c}$ and J$'$ are intra planer, intra bilayer and inter bilayer interactions, respectively, as shown in Fig. 1. The anisotropic exchange Hamiltonian becomes:

2 $$\begin{aligned} H = -\frac{1}{2}\sum _{ij}\left( J _{ab} \left( S_{i}^{x}S_{j}^{x} + S_{i}^{y}S_{j}^{y}\right) + (J' + J _{c})S_{i}^{z}S_{j}^{z}\right) \end{aligned}$$

The strength of magnetic anisotropy along a particular direction (axis or plane) is decided by the ratio of $J _{ab}$ and ($J' + J _{c}$). The exchange parameters $J'$, $J_{c}$ and $J _{ab}$ are temperature dependent as discussed later in "Phase transitions in La_1.4_Sr_1.6_Mn_2_O_7_" section. Eq. (2) is similar to the XXZ model Hamiltonian^31^.

The perovskite bilayer La$_{1.4}$Sr$_{1.6}$Mn$_{2}$O$_{7}$ (BL-LSMO-0.3) manganite belongs to a particular composition of bilayer series La$_{2-2x}$Sr$_{1+2x}$Mn$_{2}$O$_{7}$ which is a member of Ruddlesden–Popper (RP) series manganites (La, Sr)$_{m+1}$Mn$_{m}$O$_{3m+1}$ having centrosymmetric structure, where $m = 1, 2, 3,..., \infty$ implies monolayer (La, Sr)$_{2}$Mn O$_{4}$ (La$_{1-x}$Sr$_{1+x}$MnO$_{4}$), bilayer (La, Sr)$_{3}$Mn$_{2}$O$_{7}$ (La$_{2-2x}$Sr$_{1+2x}$Mn$_{2}$O$_{7}$), trilayer (La, Sr)$_{4}$Mn$_{3}$O$_{10}$ (La$_{3-3x}$Sr$_{1+3x}$Mn$_{3}$O$_{10}$)..., infinite layer (La, Sr)MnO$_{3}$ (La$_{1-x}$Sr$_{x}$MnO$_{3}$), respectively^32^. Structurally, *m* represents the number of stacked MnO$_{2}$ planes between (La, Sr)O block layers^33^. Hence, bilayer La$_{2-2x}$Sr$_{1+2x}$Mn$_{2}$O$_{7}$ is composed of an alternate stacking of two stacked MnO$_{2}$ planar layer (a magnetic conducting bilayer) and (La, Sr)$_{2}$O$_{2}$(a non-magnetic insulating) rock-salt layer along c-axis^34^, resulting Q2D layered structure as shown schematically in Fig. 1. Conducting and FM nature of the stacked MnO$_{2}$ layers are caused by double exchange (DE) interaction^35^ in presence of mixed oxidation state of Mn (Mn$^{+3}$/Mn$^{4+}$). According to the DE mechanism, electrons hop in e$_{g}$ orbitals between neighboring Mn$^{3+}$ and Mn$^{4+}$ sites with strong on-site Hund’s coupling, through O$^{2-}$ ions. This leads to enhance charge transport in the FM state when the local Mn *d*-shell spins are parallel. Thus, the hopping electrons promote FM ordering of neighboring Mn sites because they tend to preserve their spin direction. Each bilayer consists of two MnO$_{2}$ layer (2D plane) attached by Mn–O–Mn bonding perpendicular to the plane of MnO$_{2}$ layer, and form Q2D layer. These bilayers are conducting and their conducting behavior is enhanced at low temperature due to magnetic DE interaction between Mn$^{4+}$ and Mn$^{3+}$ ions while (La, Sr)$_{2}$O$_{2}$ rock-salt layers having no charge carriers, are insulating. Hence, bilayer La$_{2-2x}$Sr$_{1+2x}$Mn$_{2}$O$_{7}$ forms conducting-insulating pattern. In the layered structure of bilayer La$_{2-2x}$Sr$_{1+2x}$Mn$_{2}$O$_{7}$, some intriguing phenomena such as strong anisotropy and some other complex properties have been studied^34,36–44^. The magnetic and structural properties of bilayer La$_{2-2x}$Sr$_{1+2x}$Mn$_{2}$O$_{7}$ manganites have been studied by neutron powder diffraction in the region 0.3 $\le$ x $\le$ 1, giving a rich magnetic and structural phase diagram^45^. A small change in doping concentration in bilayer La$_{2-2x}$Sr$_{1+2x}$Mn$_{2}$O$_{7}$, induces a significant change in magnetic characteristics from uniaxial ferromagnetism (0.313 $\le$x < 0.32) to planar ferromagnetism (0.32 $\le$ x $\le$ 0.35)^46^. The magnetic structures of bilayer La$_{2-2x}$Sr$_{1+2x}$Mn$_2$O$_7$ (x $=$ 0.315)using neutron diffraction measurements have been shown to be uniaxial 2D Ising FM below 60 K, canted antiferromagnetic (CAFM) in between 60 K and 115 K, and paramagnetic (PM) above 115 K^47^. Previous reports on bilayer La$_{2-2x}$Sr$_{1+2x}$Mn$_{2}$O$_{7}$ (x= 0.3, 0.33, 0.34, 0.4, etc.) manganite perovskites claim that there is a 2D short-range (SR) FM ordering between 3D FM (T < T$_{C}$), and PM phase (T > T$_{C}$), where T$_{C}$ is transition temperature^40,48,49^. The critical behavior of cubic perovskite infinite layer manganites La$_{1-x}$Sr$_{x}$MnO$_{3}$ have been studied thoroughly for different doping concentrations^50–58^, while bilayer La$_{2-2x}$Sr$_{1+2x}$Mn$_{2}$O$_{7}$ is not much studied, the only most studied composition is La$_{1.2}$Sr$_{1.8}$Mn$_{2}$O$_{7}$ (x = 0.4)^59^. Specific heat measurement of La$_{1.2}$Sr$_{1.8}$Mn_2_O$_{7}$ shows a planar XY or 2D-Ising critical fluctuation^60^. The neutron scattering experiments^61^, have shown that there is existence of 2D Ising interaction with $\beta$ = 0.13 below T$_{C}$ = 116 K for x = 0.4 and above 116 K there is coexistence of FM and AFM clusters. Spins in one plane are canted in different direction with respect to the spins in the other plane of the bilayer. This is due to the superexchange (SE) interaction between Mn ions of these two MnO$_{2}$ planes, which results to the AFM coupling between the planes. The canting angle between two spins of different MnO$_{2}$ planes decreases with an increase in magnetic field. The critical behavior of La$_{1.2}$Sr$_{1.8}$Mn$_{2}$O$_{7}$ follows none of the standard universality classes^62^. Later on, Thanh et al. again studied the critical behavior of La$_{1.2}$Sr$_{1.8}$Mn$_{2}$O$_{7}$ and have shown that the universality class changes with the applied magnetic field^59^. In the bilayer manganites, most of the reports^49,60–63^ have shown that there exists only one transition from 2D Ising FM to PM, although AFM and FM couplings were observed after transition^47,61^. Some of these reports^47,61^ show the temperature and field dependent canting with AFM states as well as SR ordered competing AFM and FM clusters. It appears that all these works are unconnected and ambiguous because they do not provide the comprehensive view of the magnetic properties and phase transition between different phases.

Here, we show that BL-LSMO-0.3 undergoes multiple magnetic phase transitions at T$_{C1} \approx$ 101 K, T$_{C2} \approx$ 246 K and T$_{C3} \approx$ 295 K. All these transitions have been investigated by the change in entropy and their critical behavior. All three magnetic phase transitions are second order, and all four phases are 2D Ising FM (below T$_{C1}$), 2D Heisenberg CAFM (T$_{C1}$ to T$_{C2}$ and T$_{C2}$ to T$_{C3}$) and PM (above T$_{C3}$). In this context, it is worthwhile to mention that the existence of competition between different exchange interactions give rise to multiple phases with multiple phase transitions as we have shown recently in Cu$_2$OSeO$_3$^64^, which is a skyrmion host helimagnetic system.

## Experimental details

BL-LSMO-0.3 and infinite layer La$_{0.7}$Sr$_{0.3}$MnO$_{3}$ (IL-LSMO-0.3) sample were prepared by standard solid-state reaction method. High purity La$_{2}$O$_{3}$ (Sigma Aldrich 99.99%), SrCO$_{3}$ (Alfa Aesar 99.995%), and MnO$_{2}$ (Alfa Aesar 99.997%) were used as precursor. The precursors La$_{_{2}}$O$_{3}$, and SrCO$_{3}$ were pre-heated at 1000 $^{\circ }$C for 12 h and at 150 $^{\circ }$C for 12 h, respectively, to avoid any error in weight due to some expected moisture. Required stoichiometric ratio of these precursors for BL-LSMO-0.3 and IL-LSMO-0.3 were mixed homogeneously by grinding. The BL-LSMO-0.3 was calcined at 1050 $^{\circ }$C for 48 h and sintered at 1400 $^{\circ }$C for 36 h. Similarly, IL-LSMO-0.3 was calcined at 1050 $^{\circ }$C for 24 h and sintered at 1400 $^{\circ }$C for 15 h. The final step was repeated to obtain the single phase of the samples^65,66^. All the reaction process takes place in the air at ambient pressure so that samples be prepared in the proper stoichiometric ratio. The X-ray diffraction (XRD) data of the samples were collected using Rigaku X-ray diffractometer with Cu-K$_{\alpha }$ line. The high precision magnetic measurements were carried out using the physical properties measurement system (PPMS) in three different ways; (1) Field cooled (FC) temperature scans: the sample was cooled from room temperature to the desired low temperature under an external field, and temperature-dependent magnetization (M-T) data were recorded during warming in the presence of the magnetic field. (2) Zero field cooled (ZFC) temperature scans: the sample was brought at low temperature in absence of magnetic field, and then data were collected during warming by applying a magnetic field. (3) Magnetic field scans: the sample was brought at the various temperatures and held until the thermal equilibrium was reached. First quadrant magnetization (M-H) data for BL-LSMO-0.3 were collected up to 5 T in step of 10 mT from 0 to 500 mT, and then step size was increased to 200 mT for above 500 mT. Four quadrant M-H data were also collected at 10 K and 130 K to explore the different magnetic behaviors of respective phase regions.

**Figure 2:**
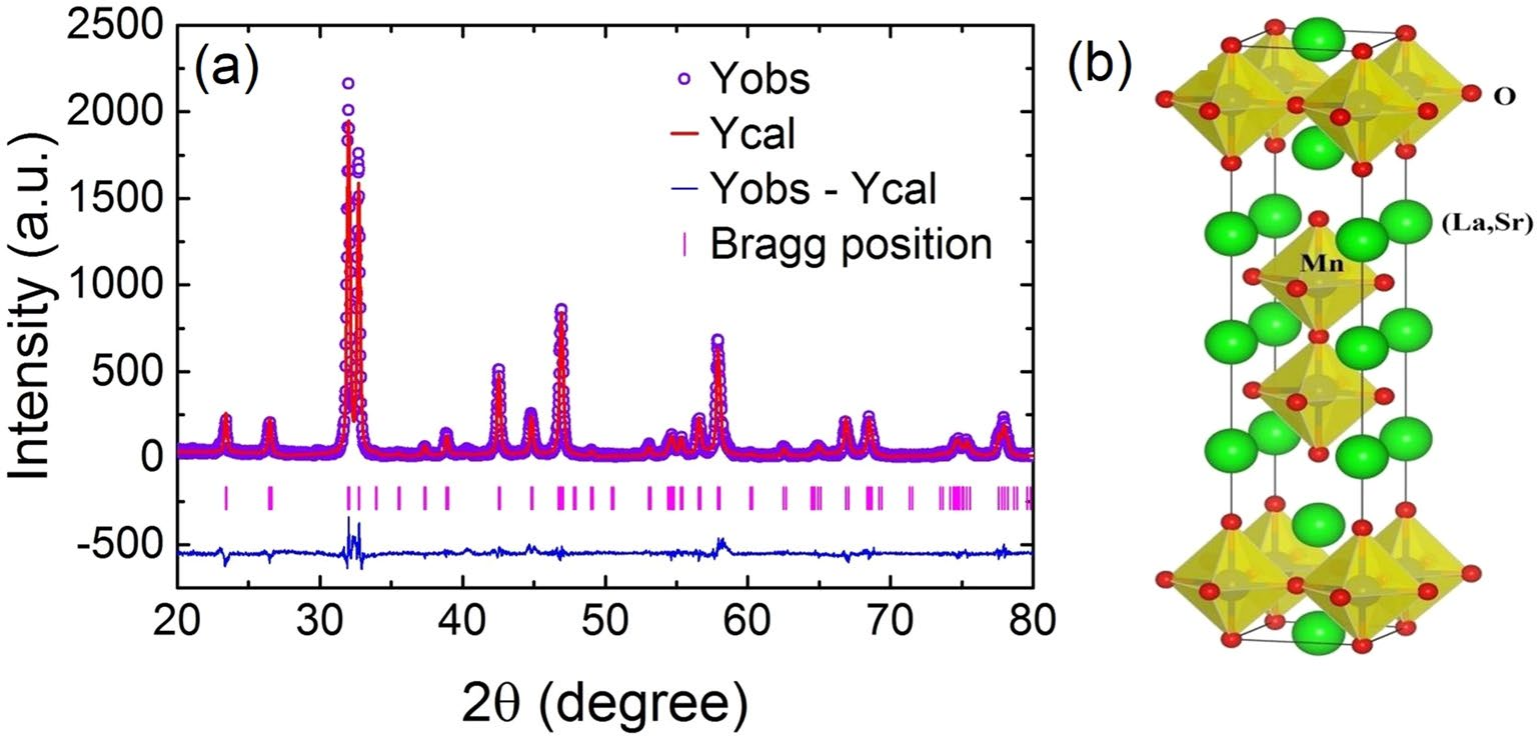
(**a**) Rietveld refinement of the room temperature XRD of La$_{1.4}$Sr$_{1.6}$Mn$_{2}$O$_{_{7}}$. Navy blue circles: experimental data. Red line: calculated pattern. Pink ticks: positions of the Bragg reflections for the main phase. Blue line: difference between the experimental and calculated patterns, and (**b**) crystal structure of the bilayer La$_{1.4}$Sr$_{1.6}$Mn$_{2}$O$_{7}$. The MnO$_{6}$ octahedrons in the crystalline bulk are denoted in yellow color, whereas different colors of the spheres represent the different atoms (red—O, light green—(La, Sr) and blue—Mn). The bilayer repeat distance is $\approx$ 9.90 Å.

## Results and discussion

### X-ray diffraction analysis

X-Ray diffraction (XRD) of the BL-LSMO-0.3 sample was used to investigate its phase purity and crystal structure. The Rietveld refinement of XRD using Fullprof software (Fig. 2a) shows that the BL-LSMO-0.3 sample crystallizes in tetragonal structure with the lattice parameters $a = b = 3.871$ Å and $c = 20.215$ Å and the *I4/mmm* space group. The value of refined parameters are shown in the Table 1. The absence of impurity peak has confirmed the single-phase formation of the BL-LSMO-0.3. The crystal structure (Fig. 2b) for our sample was constructed in axial view with the help of VESTA software using refined crystal parameters of XRD data. The unit cell consists of ten MnO$_{6}$ octahedrons, out of which two are inside, and the remaining eight are sitting at the corners of the unit cell. The in-plane sharing of O atoms with octahedron forms the MnO$_{2}$ planes. The bilayer MnO$_{2}$ planes and rock-salt layers are stacked alternately i.e., two successive MnO$_{2}$ planes are separated by one rock-salt layer and thus forming Q2D layer. The distance between two MnO$_{2}$ planes in the bilayer is $\approx$ 3.96 Å, and the bilayer repeat distance is $\approx$ 9.90 Å.

**Table 1:** Room temperature value of Rietveld refinement parameters for the La$_{1.4}$Sr$_{1.6}$Mn$_2$O$_7$.

Parameters	La$_{1.4}$Sr$_{1.6}$Mn$_2$O$_7$
Symmetry	Tetragonal
Space group	*I4/mmm*
a = b(Å)	3.871
c(Å)	20.215
V(Å$^3$)	302.939
R$_p$ (%)	16.700
R$_{wp}$ (%)	20.600
R$_F$ (%)	4.180
$\chi ^2$ (%)	1.894

### Anistropy in La$_{1.4}$Sr$_{1.6}$Mn$_{2}$O$_{7}$

**Figure 3:**
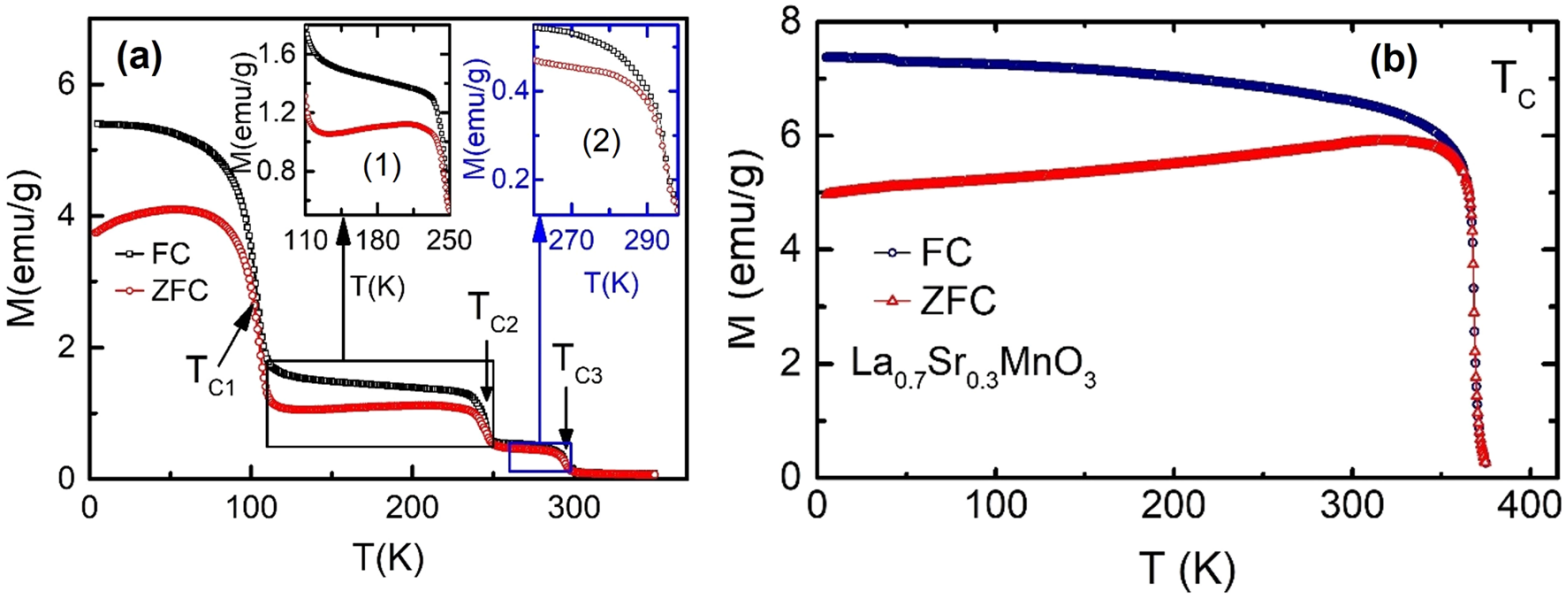
(**a**) FC and ZFC for BL-LSMO-0.3 in the temperature range 5–350 K, which shows bifurcation around all the three transitions. Inset (1) and (2) are the expanded view of FC and ZFC for T$_{C1}<$ T < T$_{C2}$ and T$_{C2}<$ T < T$_{C3}$ to see the clear bifurcation around T$_{C2}$ and T$_{C3}$, respectively. (**b**) FC and ZFC for IL-LSMO-0.3 in the temperature range 5–380 K, which shows the overlapping of FC and ZFC around and above transition.

Figure 3a shows multiple magnetic phase transitions at T$_{C1}$, T$_{C2}$ and T$_{C3}$ having significant bifurcation between FC and ZFC for BL-LSMO-0.3 while Fig. 3b indicates one transition at 368 K for IL-LSMO-0.3. Generally, if a magnetic system undergoes a magnetic phase transition, the FC and ZFC curves show a bifurcation below transition, and both curves meet near the transition point^67^. The divergence between FC and ZFC is high below transition temperature in spin glass system due to strong magnetic frustration^68^. However, divergence between FC and ZFC is always observed in magnetically ordered system, as well^67,69^, though the extent of divergence is much smaller in magnetically ordered system compared to spin glass. Ideally, there should not be any difference between magnetization under FC and ZFC in homogeneous and isotropic magnetically ordered systems. The origin of divergence in differently ordered magnetic system is not completely understood, though a number of possible mechanisms, such as magnetocrystalline anisotropy, anisotropy due to reduced dimension, competition between FM and AFM, random distribution of magnetic ions, and deformation in lattice, have been proposed^69^. Hence, whatever be the origin, any divergence between FC and ZFC in magnetically ordered systems can be related to “anisotropy”. The first transition appears around T$_{C1}$, below this temperature FC and ZFC show a large bifurcation and do not meet even above the transition. Similarly, in the case of second and third transitions, the FC and ZFC curves show a significant bifurcation, which is shown in the inset (1) and (2) of Fig. 3a. Quantitatively the anisotropy can be calculated by the relation^70,71^ K$_{an}$ $=$ (M$_{S}$ $\times$ H$_{C}$)/2, where K$_{an}$, M$_{S}$, and H$_{C}$ are anisotropy constant, saturation magnetization, and coercive field, respectively. The magnetic anisotropy energy K$_{an}$ which is responsible for symmetric hysteresis loop in M-H, exerts lattice torque on magnetization and induces the tendency to rotate the magnetization towards easy axis^71^. We have determined the K$_{an}$ for BL-LSMO-0.3 in three different regions using M$_{S}$ and H$_{C}$ and found that; K$_{an}$ $=$ 2548 J/m$^{3}$ at 10 K, K$_{an}$ $=$ 1626 J/m$^{3}$ at 130 K, and K$_{an}$ $=$ 206 J/m$^{3}$ at 300 K. Figure 3b shows the FC and ZFC curves for IL-LSMO-0.3. In contrast to the BL-LSMO-0.3, the IL-LSMO-0.3 shows a bifurcation below T$_{C}$, and the two curves (FC and ZFC) meet at T$_{C}$. We have also calculated K$_{an}$ $=$ 1002 J/m$^{3}$ at 10 K and K$_{an}$ $=$ 76 J/m$^{3}$ at 300 K for IL-LSMO-0.3 using M$_{S}$ and H$_{C}$. It may be noted that K$_{an}$ for BL-LSMO-0.3 is more than twice to that of the K$_{an}$ for IL-LSMO-0.3. This can be attributed to anisotropy due to layered structure and competition between FM and AFM states (discussed later). In Fig. 3b both FC and ZFC overlap near and above 368 K while in Fig. 3a FC and ZFC do not overlap at T$_{C1}$ and T$_{C2}$. This non-overlapping of FC-ZFC below and above transition are due to the existence of magnetic interactions at these points, as evidenced by non zero magnetic moments after T$_{C1}$ and T$_{C2}$.

### Phase transitions in La$_{1.4}$Sr$_{1.6}$Mn$_{2}$O$_{7}$

**Figure 4:**
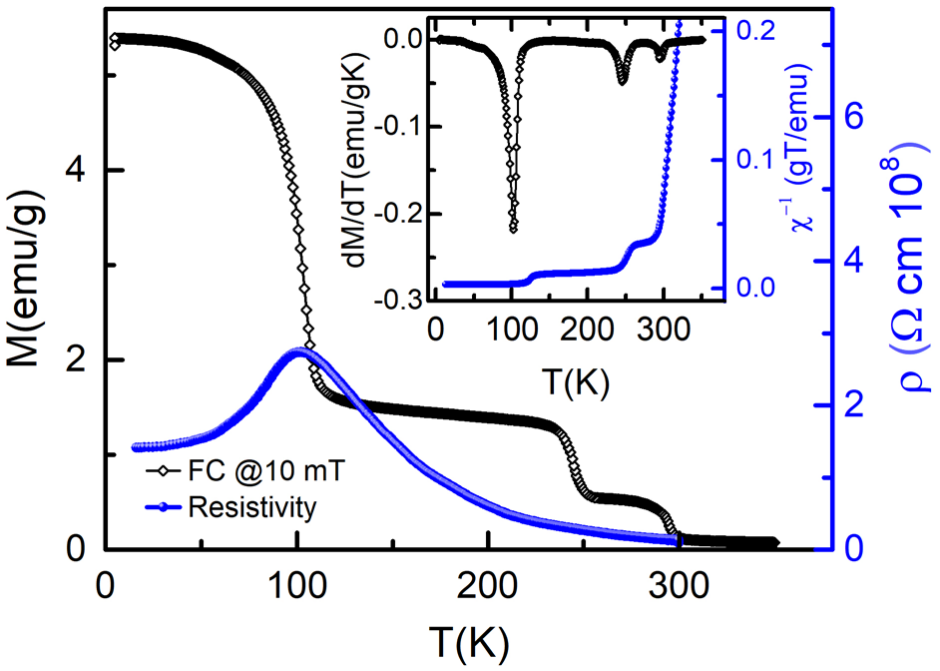
Diamond$-$ $\diamond$ represents FC of BL-LSMO-0.3 under an applied field of 10 mT. Inset is the inverse of dc susceptibility showing three transitions at $\approx$ 101 K, $\approx$ 246 K, and $\approx$ 295 K corresponding to T $_{C1}$, T $_{C2}$, and T $_{C3}$, respectively. circle$-\circ$ represents the resistivity versus temperature plot of bilayer La$_{1.4}$Sr$_{1.6}$Mn$_{2}$O$_{7}$. Metal-insulator transition temperature (T$_{MI}$) is also 101 K.

Figure 4 shows the temperature dependent magnetization in FC mode at 10 mT and resistivity data recorded in the warming mode. The inverse of magnetic susceptibility and derivative of magnetization (inset of Fig. 4) shows three transitions around T$_{C1}$, T$_{C2}$ and T$_{C3}$ i.e. there exist four magnetic phases. Generally, a magnetic transition refers to vanishing of a magnetic interaction. There are three types of magnetic interactions with varied strength in bilayer La$_{2-2x}$Sr$_{1+2x}$Mn$_{2}$O$_{7}$: (1) inter bilayers ${J^{'}}$, (2) intra bilayer $J _{c}$, i.e., inter planar in bilayer (Mn–O–Mn SE interaction perpendicular to MnO$_{2}$ plane), and (3) intra planar $J_{ab}$, i.e, Mn–O–Mn interaction in MnO$_{2}$ plane as shown in Fig. 1. Thus, these transitions at three different temperatures are due to relative strength of magnetic interactions ${J^{'}}$, $J _{c}$ and $J_{ab}$. The huge difference in T$_{C1}$, T$_{C2}$ and T$_{C3}$ implies that ${J^{'}}$ $<<$ $J _{c}$ < $J_{ab}$. Neutron scattering results^72,73^ also show that $J _{c}$ < $J_{ab}$. The deviation of $\chi ^{-1}$(T) from linearity indicates the deviation from Curie Weiss law just above T$_{C1}$, T$_{C2}$ and T$_{C3}$, which is the signature for the existence of magnetic clusters^74^. The upward and downward deviation from linearity implies the existence of the AFM and FM clusters, respectively^74^.

The nature of transition and interaction at T$_{C1}$, T$_{C2}$ and T$_{C3}$ can be investigated by detailed critical analysis such as Arrott plot, modified Arrott plots (MAPs), Kouvel–Fisher (KF), and variations in the change of entropy with temperature and magnetic fields, to reveal the appropriate universality classes. The electrical transport of BL-LSMO-0.3 has been studied by the resistivity versus temperature characteristics which shows the metal-insulator transition (MIT) at and around T$_{C1}$. The metallic behavior is related to the availability of free carriers below MIT temperature (T$_{MI}$) while above T$_{MI}$ the insulating behavior is related to the non-availability of charge carriers. Electrical transport in manganites can be explained by DE mechanism. Both the magnetic and MIT at T$_{C1}$ imply that there is an intimate relation between magnetic and transport properties, such as conducting and insulating regions are higher and lower ordered magnetic states, respectively.

### Order of phase transitions

Before doing detailed critical analysis of the BL-LSMO-0.3, it is necessary to investigate the order of all three transitions. The following techniques determine the order of phase transition: (1) Entropy analysis: i.e., the collapse of normalized entropy change versus rescaled temperature curves to a universal curve (defined below), in the vicinity of T$_{C}$ implies second-order transition, and non-collapse of these curves indicates first-order transition^75–78^, and (2) Arrott plot analysis: i.e., positive and negative slope of Arrott plot imply second- and first-order transition, respectively. Magnetic entropy change $\Delta$S$_{M}$ with temperature is required to construct the universal curve. The $\Delta$S$_{M}$ of BL-LSMO-0.3 can be computed from the isothermal M-H curve for a range of temperatures near T$_{C}$ using the Maxwell’s thermodynamic relation as^79,80^:

3 $$\begin{aligned} \Delta {S_M}(\mu {_0}H, T) = \int _{0}^{\mu {_0}H}\Bigg (\frac{\partial {M}(\mu {_0}H,T)}{\partial {T}}\Bigg )_{H}d({\mu {_0}H}), \end{aligned}$$

where, $\mu _{0}$H, and M are the applied magnetic field, and magnetization, respectively. The sign of $\Delta {S_M}$ specifies the ordering or disordering nature of the magnetic state: $\Delta {S_M} < 0$ implies magnetic ordering under applied magnetic field due to suppression of the thermal fluctuations while $\Delta {S_M}$ > 0 indicates field-induced disordering. Now, the normalized entropy change $\Delta S_{M}$(T)/$\Delta {S_M^{peak}}$ is plotted against rescaled temperature $\theta$ to confirm the order of transition. In order to construct the universal curve, all the $\Delta {S_M}$ curves are normalized by dividing their maximum value $\Delta {S_M^{peak}}$ at T$_{C}$, and then rescaled the temperature axis by choosing a reference temperature such that $\Delta {S_M}(T_{r})$/$\Delta {S_M^{peak}}$ $\ge {l}$ with $0< l < 1$^81^. The high value of *l* (close to 1) implies reference temperature is very close to T$_{C}$, may produce large numerical errors due to the limited number of data points near T$_{C}$. On the other hand, if the reference temperatures are very far from the temperature T$_{C}$ corresponding to the peak of entropy, i.e., the value of *l* is too small, other phenomena (transition) may occur in this large temperature range^79^. Hence, we choose two reference temperatures T$_{r1}$ < T$_C$ and T$_{r2}$ > T$_C$, such that $\Delta {S_M(T_{r1})}$/$\Delta {S_M^{peak}}$ = $\Delta {S_M}(T_{r2})$/$\Delta {S_M^{peak}}$ = 0.7. After obtaining two reference temperatures, the rescaled temperature $\theta$ is defined as a new temperature axis and expressed as^79^

4 $$\begin{aligned} \theta = {\left\{ \begin{array}{ll} & -(T-T{_C})/(T_{r_1}-T_{C}), \ T \le T_{C} \\ & (T-T{_C})/(T_{r_2}-T_{C}), \ T > T_{C}. \end{array}\right. } \end{aligned}$$

**Figure 5:**
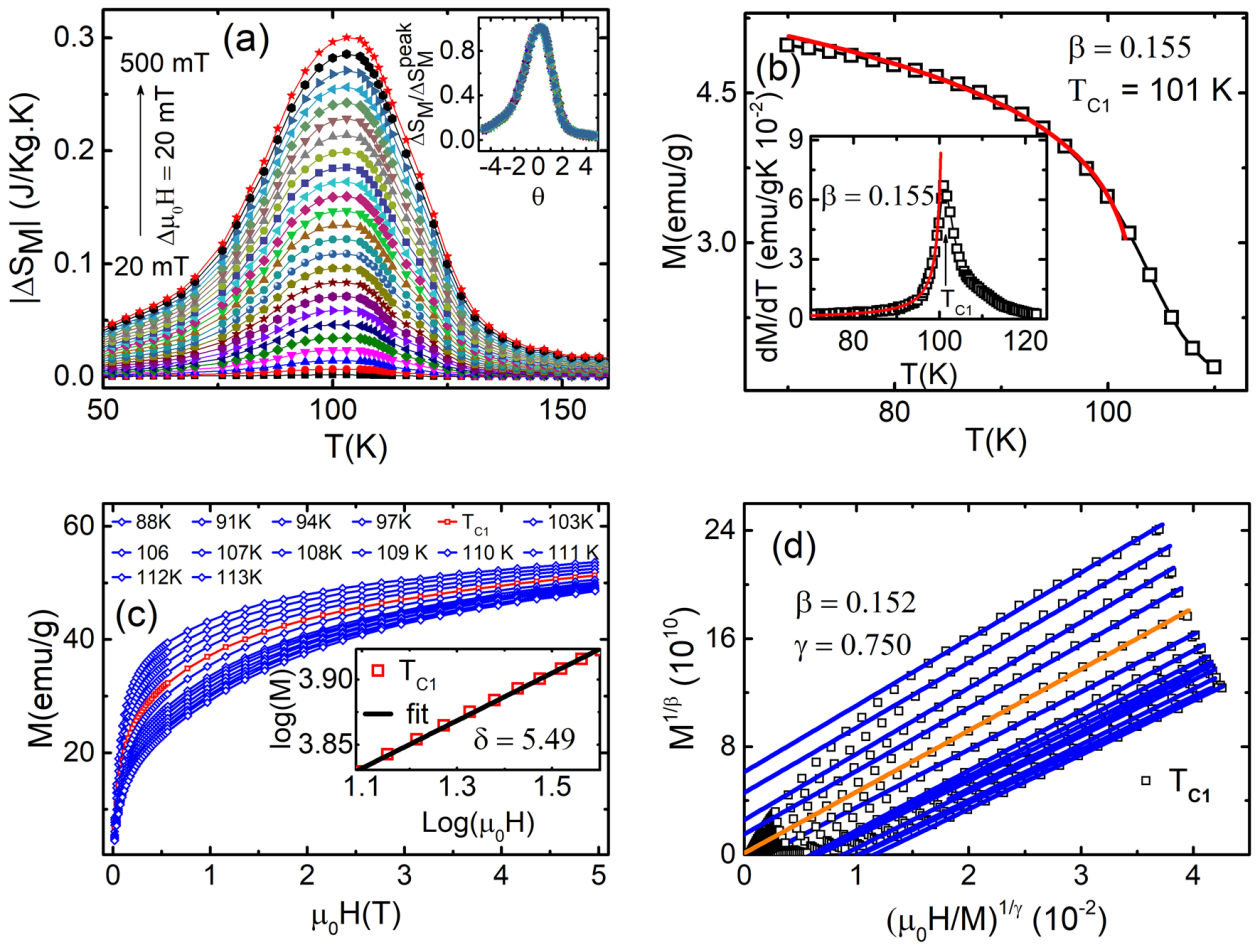
(**a**) Magnetic entropy change $\Delta$S$_{M}$ versus temperature T at different applied magnetic fields ranging from 20 to 500 mT determined by M-H curve for temperature range 50–160 K in step of 3 K, which shows a continuous non-monotonic change of $\Delta$S$_{M}$ around T$_{C1}$. Inset is normalized entropy change as a function of the rescaled temperature $\theta$ for BL-LSMO-0.3. The collapse of all the curves on a single universal curve confirms second-order phase transition. (**b**) Magnetization versus temperature around T$_{C1}$ for temperature range 5–160 K. Inset is the derivative of magnetization showing a transition at T$_{C1}$. Its fitting with Eq. (8) gives $\beta$ = 0.155. (**c**) Isothermal M-H for applied magnetic field range 0–5 T. Inset is the *log*–*log* plot of the isothermal M-H at T$_{C1}$ with fitting the log of Eq. (7), which gives the value of exponent $\delta$ = 5.49. (**d**) MAPs for the M-H of (**c**).

#### Phase transition at T$_{C1} \approx$ 101 K

Now, to find out the order of transition at T$_{C1}$ in the BL-LSMO-0.3, the change in entropy $\Delta {S_M}$ has been computed using Eq. (3) for M-H plot in temperature range from 50 K to 160 K in step of $\Delta T$ = 3 K and applied magnetic field range from 20 mT to 500 mT in step of 20 mT (Fig. 5a). This exhibits a large $\Delta {S_M}$, which may be attributed to the efficient ordering of spins^82^. The $\Delta {S_M}$ versus T curves have broad maxima and changes significantly under applied field varying from 20 mT to 500 mT. The $\Delta {S_M}$ shows non-monotonic behavior peaking at T$_{C1}$ and this is an indication of second-order phase transition. The normalized entropy change has been calculated and temperature axis was rescaled using Eq. (4) to plot $\Delta S_{M}$(T)/$\Delta {S_M^{peak}}$ versus $\theta$ curves. The collapse of all the curves on a single universal curve (inset of Fig. 5a) confirms the second-order phase transition at T$_{C1}$. The universal curve has been constructed for different magnetic fields exhibiting a second-order phase transition in the vicinity of T$_{C1}$. Since, the transition at T$_{C1}$ is second order, the critical exponents can be obtained. The critical exponents at critical points are determined by divergence of magnetic parameters like correlation length $\xi = \xi _0|(T-T_C)/T_C|^{-\nu }$, the spontaneous magnetization $M_{S}$(T), and the isothermal magnetization M-H at T$_{C}$. The spontaneous magnetization $M_{S}$ defined below T$_{C}$, the inverse of magnetic susceptibility at zero field $\chi ^{-1}_{0}$ defined above T$_{C}$ and the isothermal magnetization M-H at T$_{C}$ are associated with the critical exponents $\beta$, $\gamma$ and $\delta$, respectively. Their behavior follows the following relations, called critical scaling equations^83–85^:

5 $$\begin{aligned}&{M_S(T) = M_0(-\varepsilon )^\beta }; \ {\varepsilon<0},\ {T< T_C}, \end{aligned}$$

6 $$\begin{aligned}&{\chi ^{-1}_{0}(T) \propto (\varepsilon )^\gamma }; \ {\varepsilon <0},\ {T > T_C}, \end{aligned}$$

and

7 $$\begin{aligned} {M = D(\mu _{0}H)^{1/\delta }; \ {\varepsilon = 0}}, \ {T = T_C}. \end{aligned}$$

The derivative of Eq. (5) is

8 $$\begin{aligned} {dM_S(T)/dT = -\beta M_0(-\varepsilon )^{\beta -1}}; \ {\varepsilon<0},\ {T< T_C}, \end{aligned}$$

**Figure 6:**
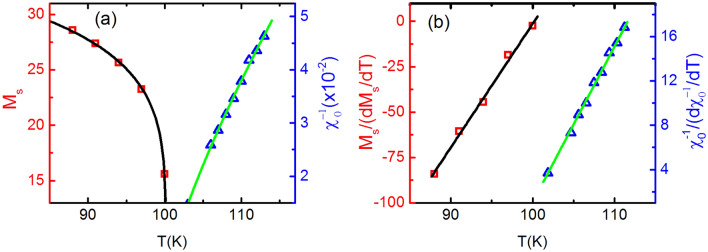
(**a**) The square symbol represents the $M_{S}$ versus T plot and the solid curve is its fitting with Eq. (5) which results into $\beta =$ 0.148. The triangle symbol represents the $\chi ^{-1}_{0}$ versus T plot while solid curve passing through it, is its fitting with Eq. (6) which yields $\gamma$ = 0.761. (**b**) The square symbol is the $M_{S}/(dM_{S}/dT)$ versus T plot and the solid curve is its fitting with Eq. (10) which results into $\beta =$ 0.147. The triangle is the $\chi ^{-1}_{0}/(d\chi ^{-1}_{0}/dT)$ versus T plot while solid curve is its fitting with Eq. (11) which yields $\gamma =$ 0.763.

**Figure 7:**
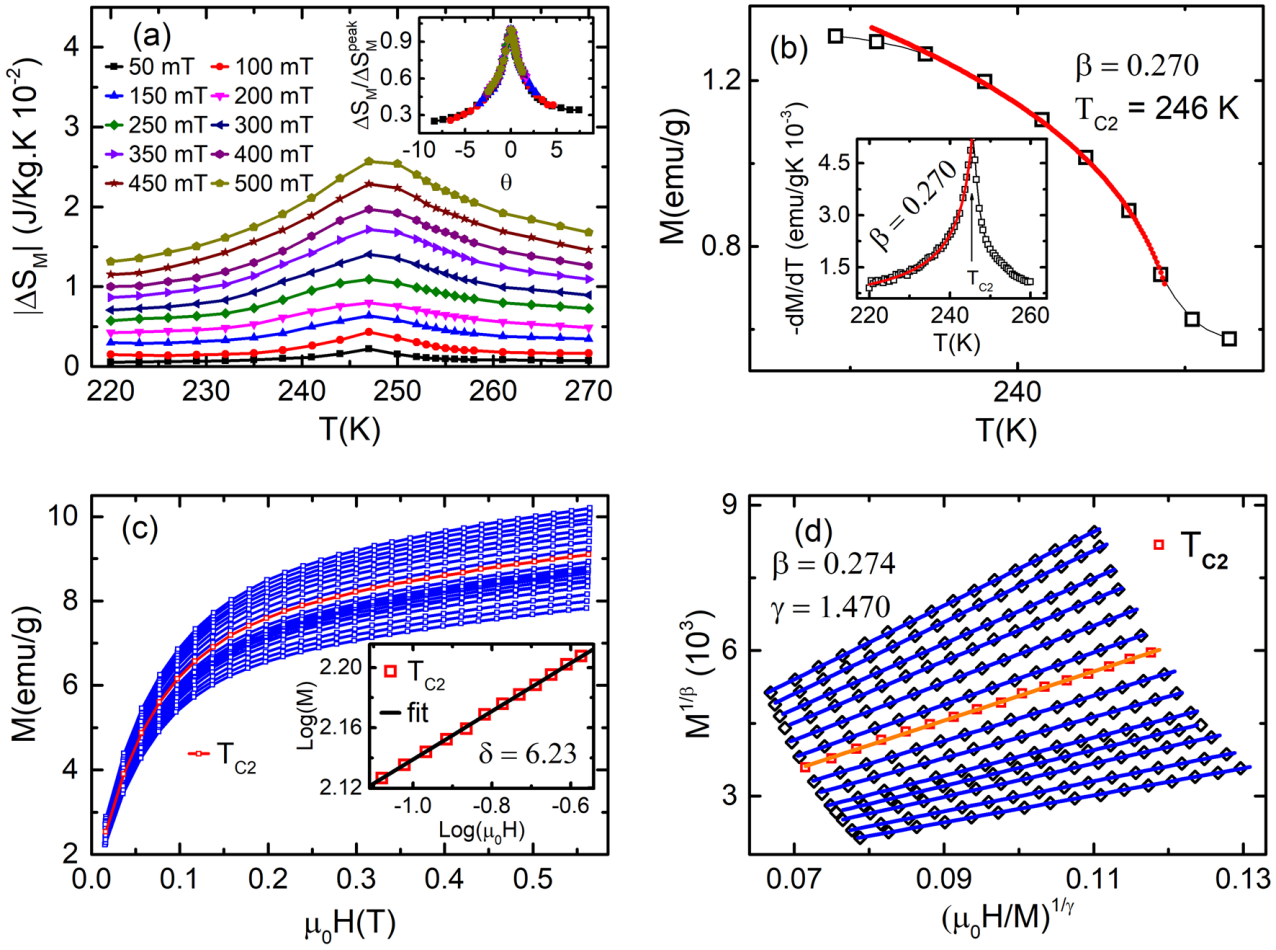
(**a**) Magnetic entropy change $\Delta$S$_{M}$ versus temperature T at different applied magnetic fields varying from 50 to 500 mT, plotted using M-H curve of (**c**). This shows a continuous non-monotonic change of $\Delta$S$_{M}$ around T$_{C2}$. Inset is normalized entropy change as a function of $\theta$. All the curves collapse on a single universal curve for the second-order phase transition. (**b**) M-T around T$_{C2}$ for temperature range 220–280 K. Inset is the derivative of magnetization having a transition at T$_{C2}$. Its fitting with Eq. (8) gives $\beta$ = 0.270. (**c**) M-H for applied magnetic field ranging from 0 to 0.6 T in the temperature range 223–270 K in the step of 3 K. Inset is the *log*–*log* plot of the M-H at T$_{C2}$ with fitting the log of Eq. (7), which gives the value of exponent $\delta$ = 6.230. (**d**) MAPs of BL-LSMO-0.3 show linear behavior in higher applied field, with the plot at T$_{C2}$ seems to passing through the origin.

**Figure 8:**
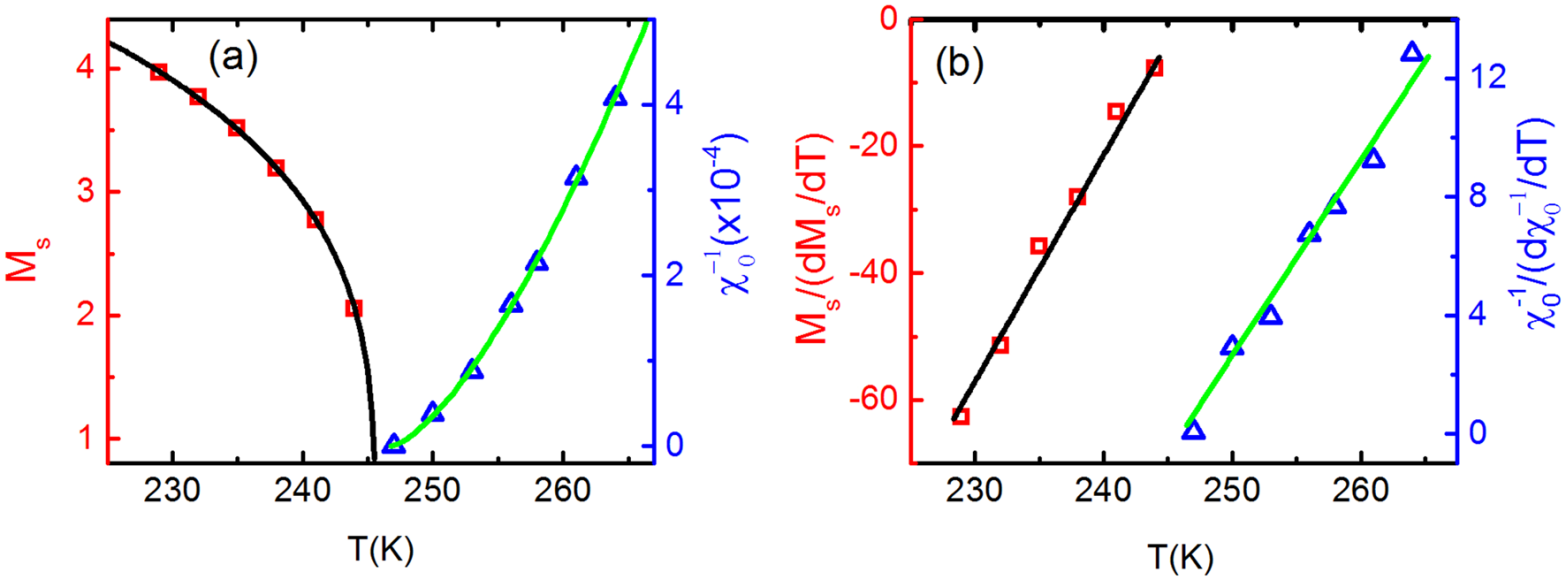
(**a**) The square symbol represents $M_{S}$ versus T plot and the solid curve is its fitting with Eq. (5) which results into $\beta =$ 0.278. The triangular symbol represents the $\chi ^{-1}_{0}$ versus T plot while solid curve is its fitting with Eq. (6) which yields $\gamma =$ 1.48. (**b**) The squares are the $M_{S}/(dM_{S}/dT)$ versus T plot and the solid curve is its fitting with Eq. (10) which results into $\beta =$ 0.280. The blue triangle is the $\chi ^{-1}_{0}/(d\chi ^{-1}_{0}/dT)$ versus T plot while green curve is its fitting with Eq. (11) which yields $\gamma =$ 1.511.

where $M_0$, and *D* are critical amplitudes. The critical exponent $\beta$ can be obtained by fitting Eq. (5) with M-T plot (Fig. 5b) at zero field or very weak applied magnetic field. But, due to the existence of finite magnetic moment after transition, fitting of M-T data with Eq. (5) becomes difficult. Hence, one has to find out the value of $\beta$ by fitting the derivative of M-T, which varies with T$^{\beta -1}$ below T$_{C}$. The fitting of the M-T derivative with Eq. (8) (inset of Fig. 5b) has yielded $\beta = 0.155$. The value of critical exponent $\delta$ is determined by fitting the log of Eq. (7) i.e. straight line with *log*–*log* plot of M-H at T$_{C}$. This results $\delta$ = 5.490 for M-H at T$_{C1}$ (inset of Fig. 5c). Using Widom scaling law $\gamma / \beta + 1 = \delta$ for $\beta = 0.155$ and $\delta = 5.490$, the value of $\gamma = 0.690$ is obtained. Universally, the critical exponents $\beta$ and $\gamma$ should follow the Arrott-Noakes equation of state^86^

9 $$\begin{aligned} (\mu _{0}H/M)^{1/\gamma } = (T-T_C)/T_C+(M/M_1)^{1/\beta }, \end{aligned}$$

in an asymptotic region $|\varepsilon |$ < 0.1, where $M_1$ is the material constant. Usually, if a system exhibits the long-range ordering, then T$_{C}$ and the critical exponents can be determined by the Arrott plot of magnetic isotherms at various temperatures defined for the mean-field model ($\beta$ = 0.5 and $\gamma$ = 1). The Arrott plot states that if the system exhibits long-range ordering, then $M^{1/\beta }$ versus $(\mu _{0}H/M)^{1/\gamma }$ will show a set of parallel straight lines, and the T$_{C}$ line should pass through the origin. Transforming M-H plot (Fig. 5c) in the form of Eq. (9) so that $M^{1/\beta }$ versus $(\mu _{0}H/M)^{1/\gamma }$ plots become parallel at higher field known as MAPs (Fig. 5d), results the values of $\beta$ and $\gamma$. From MAPs for our sample around T$_{C1}$, the value of $\beta$ and $\gamma$ are 0.152 and 0.750 respectively. Using Widom scaling law $\gamma / \beta + 1 = \delta$, the value of $\delta$ is found to be 5.930.

The most reliable and accurate value of critical exponents $\beta$ and $\gamma$ are generally determined by the KF method using the following equations^87^:

10 $$\begin{aligned} \frac{M_{S}(T)}{dM_{S}(T)/dT} = \frac{T-T_{C}}{\beta }, \end{aligned}$$

and

11 $$\begin{aligned} \frac{\chi ^{-1}_{0}(T)}{d\chi ^{-1}_{0}(T)/dT} = \frac{T-T_{C}}{\gamma }. \end{aligned}$$

In this method, the slope of $M_{S}/(dM_{S}/dT)$ versus T and $\chi ^ {-1}_{0}/(d\chi ^{-1}_{0}/dT)$ versus T gives the value of critical exponents $\beta$ and $\gamma$, respectively. Now, the $M_{S}$ and $\chi ^{-1}_{0}$ at different temperatures has been determined by using MAP for transition around T$_{C1}$ (Fig. 5d). The $M_{S}$ versus T and $\chi ^{-1}_{0}$ versus T has been plotted and then from these plots, the $M_{S}/(dM_{S}/dT)$ versus T and $\chi ^ {-1}_{0}/(d\chi ^{-1}_{0}/dT)$ versus T have been constructed as shown in Fig. 6. The fitting of Eqs. (10) and (11) with $M_{S}/(dM_{S}/dT)$ versus T and $\chi ^{-1}_{0}/(d\chi ^{-1}_{0}/dT)$ versus T yields the value of exponents $\beta =$ 0.147 and $\gamma =$ 0.763, respectively. The value of $\delta =$ 6.190 can be calculated by Widom scaling law. The values of $\beta$ (0.155, 0.152 and 0.147) determined by three different techniques are closest to the theoretical value of $\beta =$ 0.125 for SR 2D Ising model^88^, which suggests that BL-LSMO-0.3 below T$_{C1}$ is SR 2D Ising ferromagnet.

#### Phase transition at T$_{C2} \approx$ 246 K

The order of transition at T$_{C2}$ has been investigated by entropy analysis using M-H plot and employing Eqs. (3) and (4) . The $\Delta {S_M}$ versus temperature plot around T$_{C2}$ (Fig. 7a) shows very weak and broad maxima, indicating that magnetic ordering is affected very weakly with temperature and applied external magnetic field. The $\Delta S_{M}$(T)$/$ $\Delta {S_M^{peak}}$ versus $\theta$ curves at different magnetic field collapse on a single universal curve. This universal characteristic of BL-LSMO-0.3 at T$_{C2}$ (inset of Fig. 7a) confirms second order transition. Now, the critical scaling around T$_{C2}$ has been carried out to determine the value of critical exponents, using the scaling Eqs. (8) and (7) . Further, from the fitting of Eq. (5) with MT and Eq. (8) with derivative of MT (inset of Fig. 7b), the value of $\beta$ has been found to be 0.270. Similarly, fitting the log of Eq. (7) with *log*–*log* plot of M-H at T$_{C2}$ (inset of Fig. 7c) yields $\delta =$ 6.230. And the Widom scaling law, $\gamma / \beta + 1 = \delta$, for the value of these exponents gives $\gamma$ = 1.410. The value of critical exponents for transition at T$_{C2}$ has been, also, determined by using Eq. (9) to the M-H plot, based on the Arrott plots method: choose the value of $\beta$ and $\gamma$ so that the curve at T$_{C2}$ becomes straight line and this should pass through the origin. Curves at temperatures other than T$_{C2}$ should be parallel to the curve at T$_{C2}$ under higher magnetic fields or for a magnetic field range. Figure 7d is the MAPs for $\beta =$ 0.274 and $\gamma =$ 1.470. The use of Widom scaling law yields $\delta =$ 6.440. Futhermore, the $M_{S}$ and $\chi ^{-1}_{0}$ at different temperatures have been determined by using MAP for transition around T$_{C2}$ (Fig. 7d). The $M_{S}$ versus T and $\chi ^{-1}_{0}$ versus T have been plotted and then from these plots, the $M_{S}/(dM_{S}/dT)$ versus T and $\chi ^{-1}_{0}/(d\chi ^{-1}_{0}/dT)$ versus T are constructed as shown in Fig. 8. The fitting of Eq. (10) and Eq. (11) with $M_{S}/(dM_{S}/dT)$ versus T and $\chi ^{-1}_{0}/(d\chi ^{-1}_{0}/dT)$ versus T has resulted the value of exponents $\beta =$ 0.280 and $\gamma =$ 1.511, respectively. The value of $\delta =$ 6.396 can be determined by Widom scaling. The value of $\beta =$ 0.280 for our sample is very close to the value of $\beta$ observed for SR 2D Heisenberg model^23–25^. Hence , the transition at T$_{C2}$ is second order and the BL-LSMO-0.3 behave like SR 2D Heisenberg magnet between T$_{C1}$ and T$_{C2}$.

**Figure 9:**
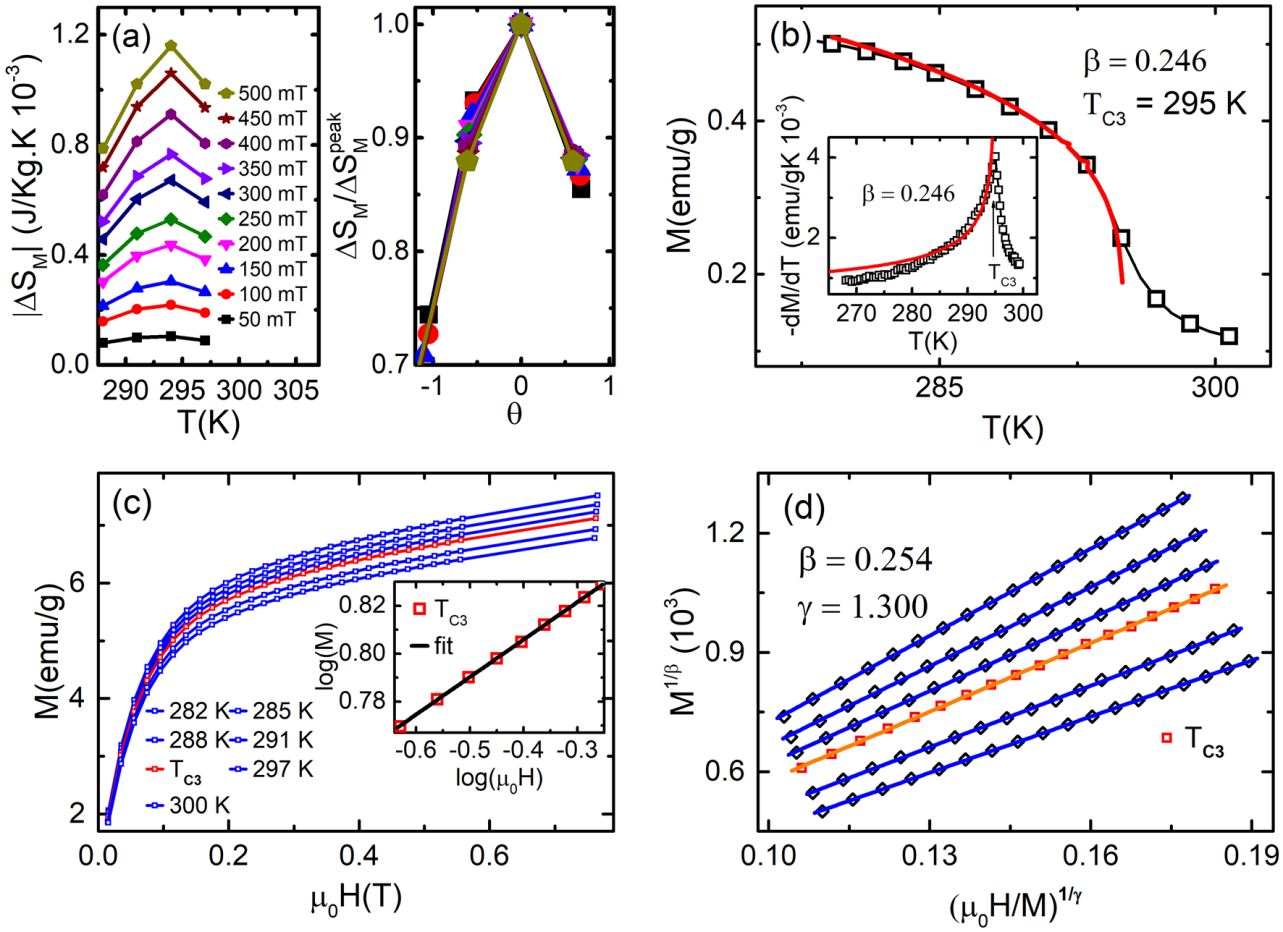
(**a**) Magnetic entropy change $\Delta$S$_{M}$ versus T at different applied magnetic fields varying from 50 to 500 mT, plotted using M-H curve of (**c**). This shows a continuous non-monotonic change of $\Delta$S$_{M}$ around T$_{C3}$. Inset is normalized entropy change as a function of the rescaled temperature $\theta$. All the curves almost collapse on a single universal curve for the second-order phase transition. (**b**) MT around T$_{C3}$ for temperature range 290–300 K. Inset is the derivative of MT which fitting with Eq. (8) gives the value of exponent $\beta$ = 0.246. (**c**) The isothermal M-H for applied field from 0 to 0.75 T. Inset is the *log*–*log* plot of the isothermal M-H at T$_{C3}$ with fitting the log of Eq. (7), which gives the value of exponent $\delta =$ 6.4. (**d**) MAPs of BL-LSMO-0.3 shows linear behavior in higher applied field with the plot at T$_{C3}$ seems to pass through the origin.

**Figure 10:**
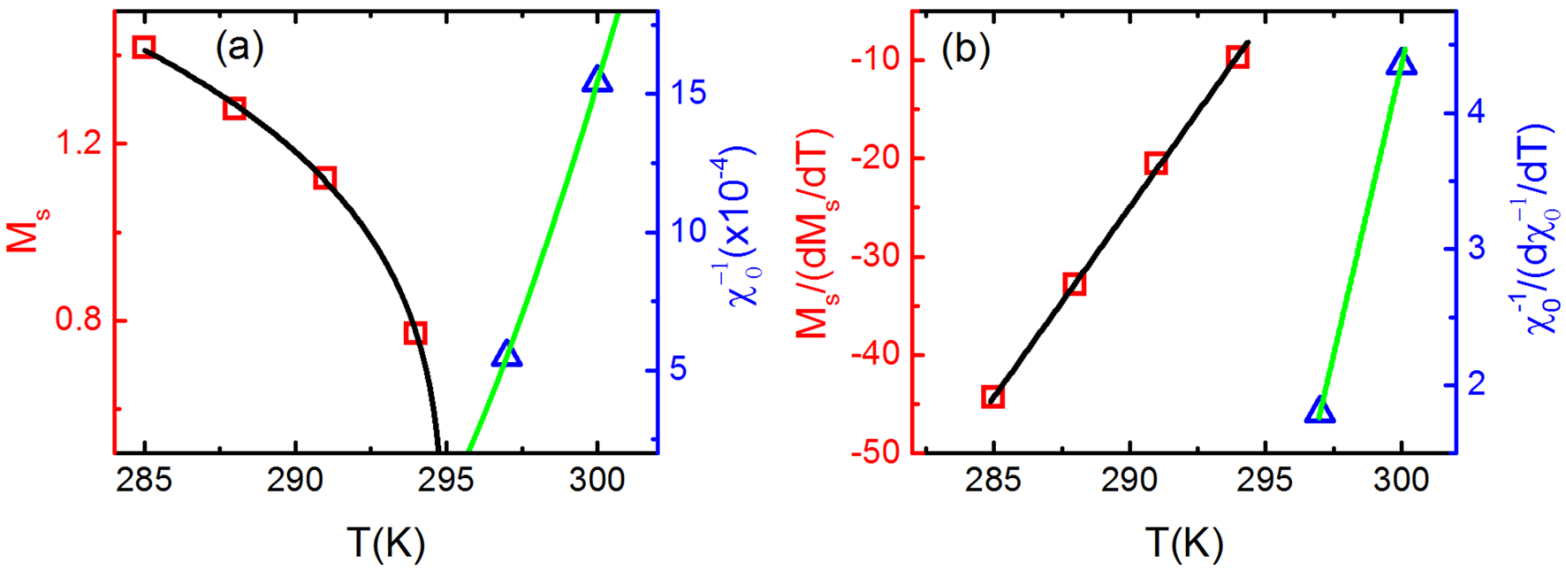
(**a**) The square symbol represents the $M_{S}$ versus T plot and the solid curve is its fitting with Eq. (5) which results into $\beta =$ 0.253. The triangular symbols corresponds to the $\chi ^{-1}_{0}$ versus T plot while solid curve is its fitting with Eq. (6) which yields $\gamma =$ 1.23. (**b**) The square symbol is representing the $M_{S}/(dM_{S}/dT)$ versus T plot and the solid curve is its fitting with Eq. (10) which results into $\beta =$ 0.258. The triangular symbol represents the $\chi ^{-1}_{0}/(d\chi ^{-1}_{0}/dT)$ versus T plot while solid curve is its fitting with Eq. (11) which yields $\gamma =$ 1.170.

#### Phase transition at T$_{C3} \approx$ 295 K

It is difficult to determine the order of transition at T$_{C3}$ by entropy analysis due to very small variation in entropy with temperature and applied field as shown in Fig. 9a. Nevertheless, the corresponding $\Delta S_{M}$(T)$/$ $\Delta {S_M^{peak}}$ versus $\theta$ curves (inset of Fig. 9a) collapse on single curve, confirming second order phase transition. However, the order of transition at T$_{C3}$ may also be identified by using MAPs or Arrott plot. The positive slopes of curves plotted by employing Eq. (9) implies second order transition at T$_{C3}$. Scaling analysis of Eqs. (5), (8) and (7) , for MT, derivative of MT (inset of Fig. 9b) and M-H at T$_{C3}$ (inset of Fig. 9c) yield $\beta =$ 0.246 and $\delta =$ 6.4, respectively. Widom scaling law, gives $\gamma =$ 1.33. Now, MAPs constructed (Fig. 9d) for M-H in (Fig. 9c), yield the values of critical exponents $\beta =$ 0.254 and $\gamma =$ 1.3. The substitution of value for these exponents $\beta$ and $\gamma$ in Widom law, $\gamma / \beta + 1 = \delta$, gives $\delta =$ 6.1. So, we have observed that both critical scaling and MAPs are providing nearly the same value of critical exponents at T$_{C3}$. Further, the best value of critical exponents $\beta$ and $\gamma$ are determined using KF method as follows: the $M_{S}$ and $\chi ^{-1}_{0}$ at different temperatures have been determined by using MAP for transition around T$_{C3}$ (Fig. 7d). The $M_{S}$ versus T and $\chi ^{-1}_{0}$ versus T have been plotted and then from these plots, the $M_{S}/(dM_{S}/dT)$ versus T and $\chi ^{-1}_{0}/(d\chi ^{-1}_{0}/dT)$ versus T have been constructed as shown in Fig. 10. The fitting of Eqs. (10) and (11) with $M_{S}/(dM_{S}/dT)$ versus T and $\chi ^{-1}_{0}/(d\chi ^{-1}_{0}/dT)$ versus T yield the value of exponents $\beta =$ 0.258 and $\gamma =$ 1.170, respectively. The value of $\delta =$ 5.535 can be easily determined by Widom scaling. The value of $\beta =$ 0.258 is closest to the the SR 2D Heisenberg^23–25^ i.e. the spins interact following 2D Heisenberg interaction, which is responsible for non-zero magnetization in the temperature range T$_{C2}$ to T$_{C3}$. Thus, the transition at T$_{C3}$ is second order and spin-spin exchange interaction is SR 2D Heisenberg type. The difference in SR 2D Heisenberg for temperature range T$_{C1}$ to T$_{C2}$ and T$_{C2}$ to T$_{C3}$, is the negligible value of inter-planar interaction, J$_c$, as discussed before.

**Figure 11:**
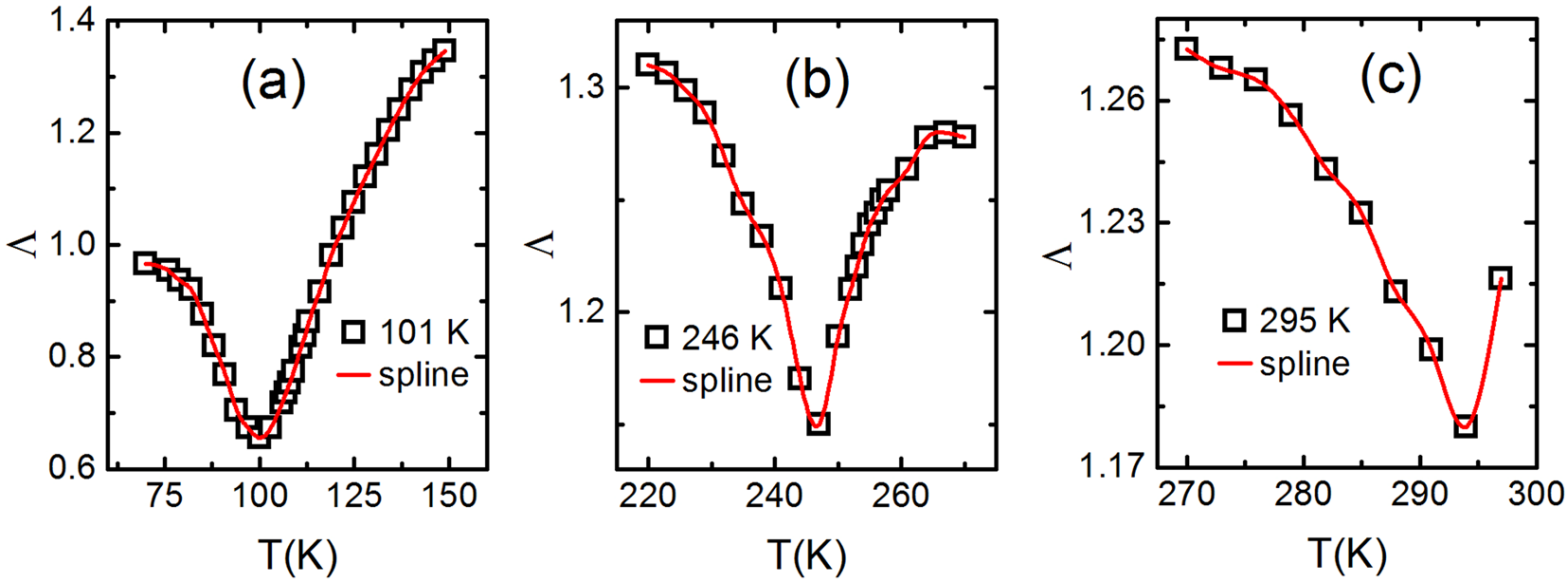
$\Lambda$ versus T plots at T$_{C1}$, T$_{C2}$ and T$_{C3}$: (**a**) The black rectangle is $\Lambda$ versus T plot around 101 K in the temperature range 70–150 K and the red curve is the spline for eye guide. (**b**) The black rectangle is $\Lambda$ versus T plot around 246 K in the temperature range 220–270 K and the red curve is the spline for eye guide. (**c**) The black rectangle is $\Lambda$ versus T plot around 295 K in the temperature range 270–297 K and the red curve is the spline fit guide to the eye.

**Figure 12:**
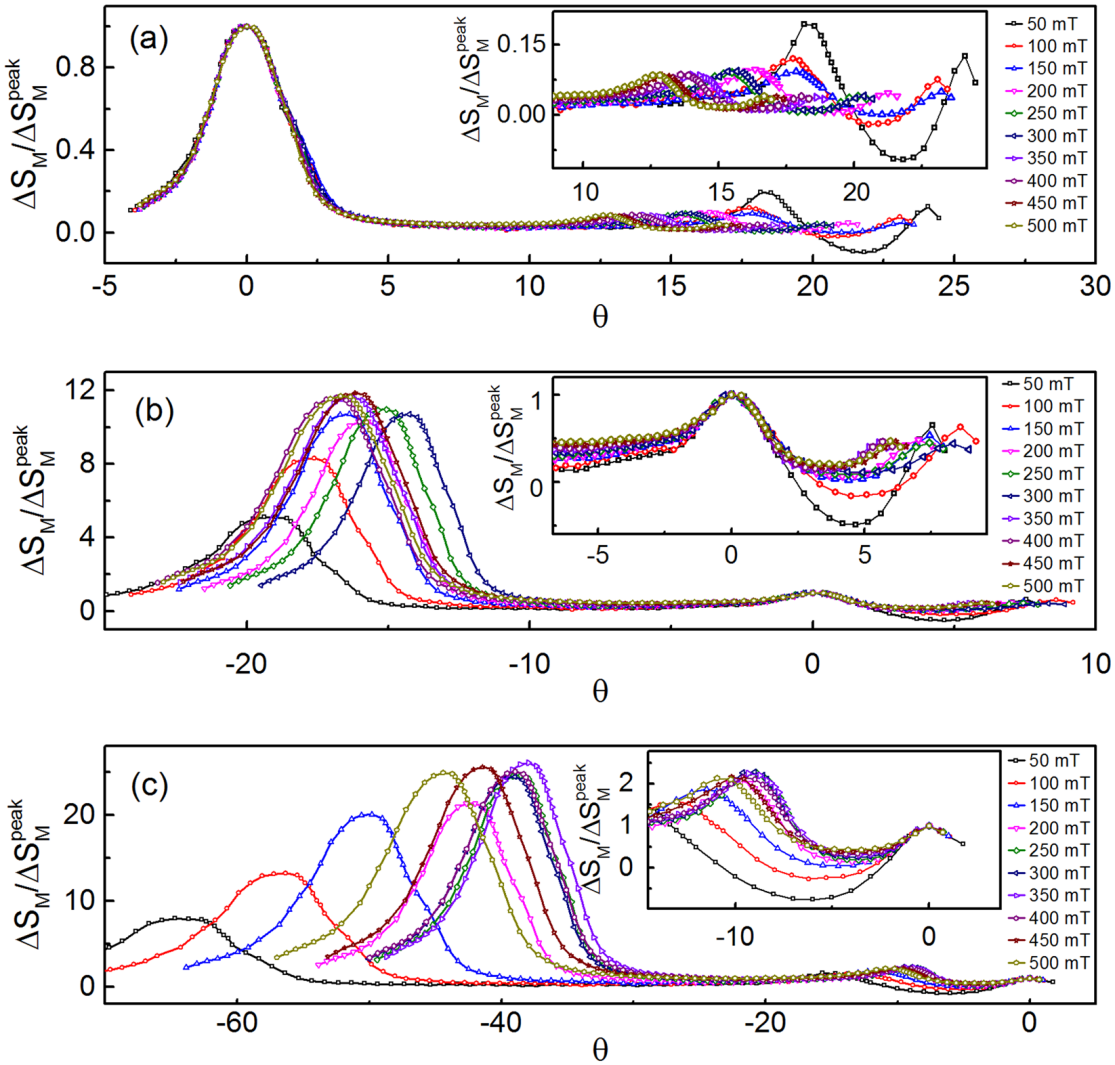
Scaling around all the three points using universal curves for field range 50–500 mT in step of 50 mT: (**a**) the scaling around 101 K for entire temperature range 50–297 K shows overlaping of universal curves for all fields however, the second (246 K) and third transition (295 K) points are shifted towards the first transition point. This effect is clearly seen in the inset. Therefore, second order transition is clearly observed at 101 K with almost no effect of other transitions. (**b**) The universal curves overlapping around 246 K for all fields are an indication of second order transition. A very small effect of other transitions are observed as shown in the inset. (**c**) The universal curves are well scaled around 295 K with a small effect of second transition is clearly seen in the inset. Hence, all the three transitions are deconvoluted very well due to significant separation between two transition points.

**Figure 13:**
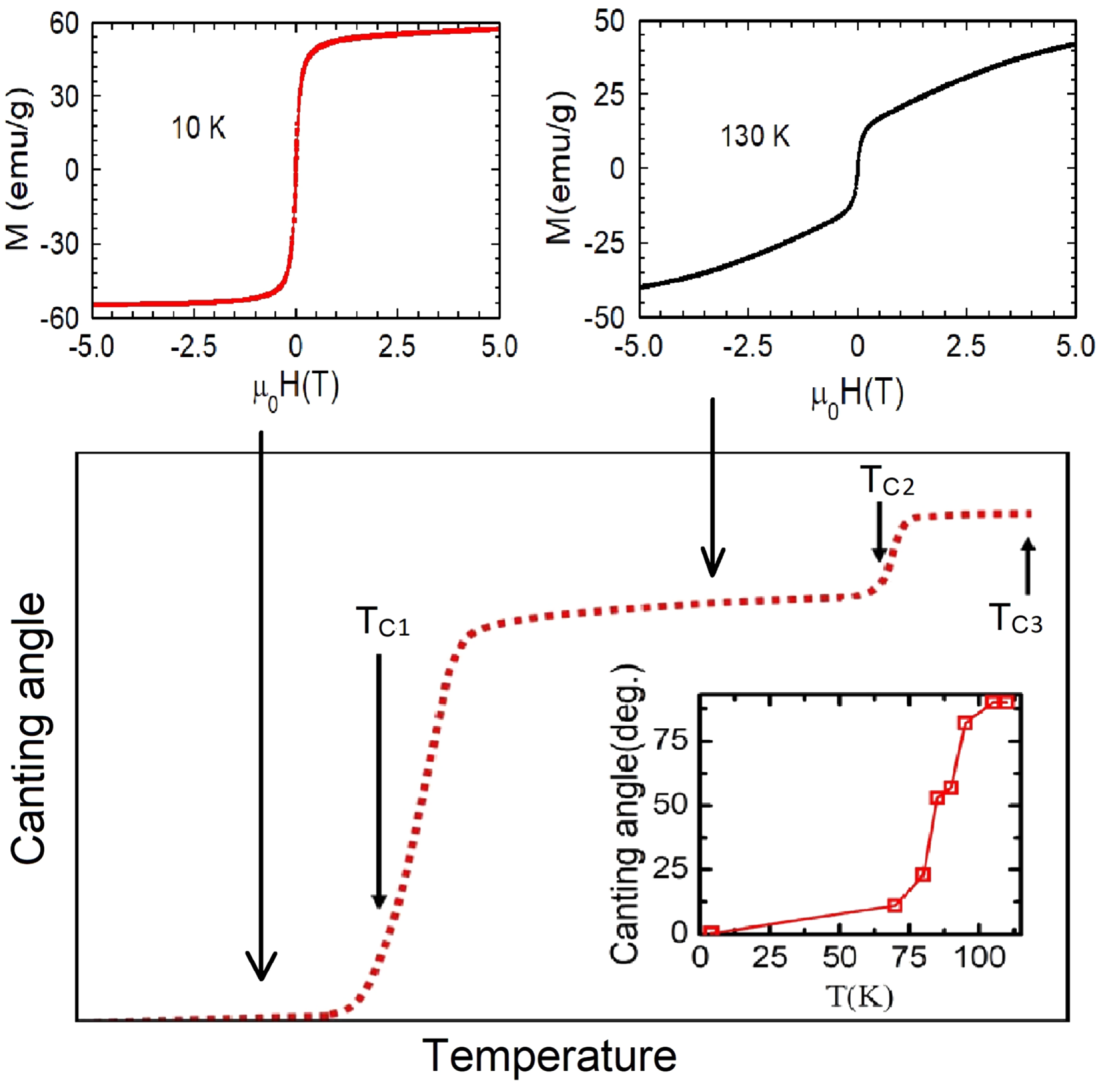
M-H at 10 K (no signature of canting) and at 130 K (canting is clearly observed). Schematic representation of canting angle versus temperature for BL-LSMO-0.3 at normal pressure. Canting angle changes uniformly in a particular phase, however, at and around transition points the canting angle changes fast. Inset is the canting angle with temperature at normal pressure for BL-LSMO-0.315^47^.

### Deconvolution of all three magnetic phases

The BL-LSMO-0.3 shows three second order phase transitions at T$_{C1}$, T$_{C2}$ and T$_{C3}$ in which first and second transition points are far separated and, second and third transition points are also separated, significantly. When transition temperatures are very close, there is effect of each phase on the other(s) and the effect is minimum for low field. Using entropy universal curve of each phase, the response of that phase for any magnetic field can be found^89^. Therefore, it is required to deconvolute the response of each phase by using the universal curve or scaled entropy curves corresponding to the weaker magnetic fields. Deconvolution of the phases are performed by two different techniques^89^: (1) assuming the power law behavior $\Delta S_{M}$ $\propto$ $H^{\Lambda }$ i.e. $\Lambda$ versus T plot, and (2) the field dependence of $\Delta S^{peak, i}$ and reference temperatures $T_{r, i}$ i.e. $\Delta S_{M}$ versus $\theta$ , where $\theta$ is determined by Eq. (4).

For BL-LSMO-0.3, the values of parameter $\Lambda$ at different temperatures have been determined by log–log plot of $\Delta S_{M}$ $\propto$ $H^{\Lambda }$ relation^90^. The plots of $\Lambda$ versus T around T$_{C1}$, T$_{C2}$ and T$_{C3}$ are shown in Fig. 11. The minima in $\Lambda$ around T$_{C1}$, T$_{C2}$ and T$_{C3}$ confirm the existence of three separate magnetic phase transitions. The deconvolution of phases employing universal curves $\Delta S_{M}/\Delta S^{peak}_{M}$ versus $\theta$ has been performed for field range 50–500 mT in step of 50 mT as shown in Fig. 12. In Fig. 12a, the universal curves at different fields overlap around first transition while peaks around other transitions move towards the first transition as the magnetic field increases. There is almost no effect on first transition due to others. Similarly, in Fig. 12b, c the universal curves overlap around second and third transition points while others come close to these points as the magnetic field increases. Of course, the two transitions come closer with increasing field but do not overlap for given field range because transitions are well separated. Thus, all the three transitions are clearly deconvoluted i.e. the three transitions in the BL-LSMO-0.3 are clearly observed.

## Discussion

The crystallographic analysis shows that BL-LSMO-0.3 is stabilized in Q2D centrosymmetric structure as shown in Fig. 1. This structural anisotropy facilitates the spins of Mn ion to orient perpendicular to its surface at low temperature^91,92^ and the material is stabilized in FM state. Structural anisotropy in our sample may induce anisotropic change in O-Mn bond length (d$_{O-Mn}$) with temperature in octahedrons of unit cell^93^ and this is responsible for the formation of polarons. All the three magnetic transitions (Fig. 4) have been shown to be second order by entropy analysis and Arrott plot analysis. These transitions can be explained due to vanishing of $J'$, $J_{c}$ and $J_{ab}$ interactions in the bilayer at T$_{C1}$, T$_{C2}$ and T$_{C3}$, respectively. The anisotropies observed (see in anisotropy section) support the existence of 2D magnetic interaction. The magnetic anisotropy due to exchange interaction for our sample may be explained by Eq. (2) as follows: below T$_{C1}$, $J'$ leads to align the spins along z-axis resulting Ising Hamiltonian, i.e., $J' \ne 0$ but for T$_{C1}$ T< T$_{C2}$, $J'\sim 0$, $J _{c}\ne 0$ and $J_{ab} \ne 0$. For T$_{C2}$  < T < T$_{C3}$, $J'\sim 0$, $J _{c} \sim 0$, and $J_{ab} \ne 0$, which leads to anisotropic Heisenberg Hamiltonian. Transition at T$_{C1}$: a report based on neutron scattering has shown that $J _{c}$ < $J_{ab}$^72,73^. So, the $J'$ would have negligible value because bilayers are more separated than that of the planes in the bilayer. Hence, the negligible value of $J'$ cannot explain pronounced transition at T$_{C1}$. However, the magnetic and resistivity transition at T$_{C1}$ may be explained by the creation or destruction of polarons with temperature^94,95^ because formation of polarons reduces charge carriers and their flow as well as decreases DE interaction between Mn$^{3+}$ and Mn$^{4+}$.

**Figure 14:**
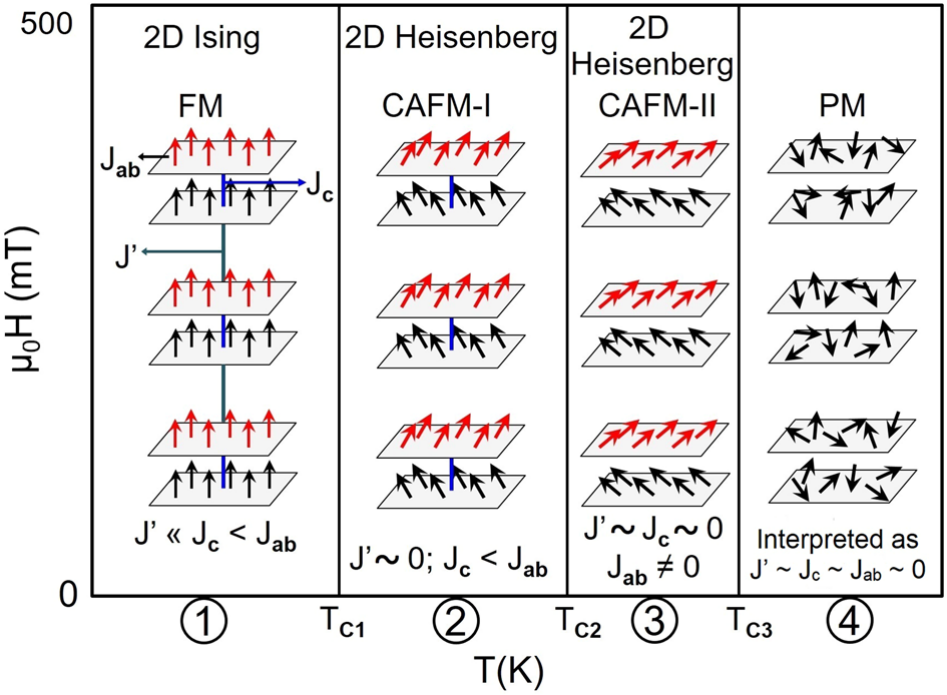
Schematic magnetic phase diagram: below T$_{C1}$, there exists high magnetic anisotropy normal to the plane of bilayer and behaving as 2D Ising FM. In between T$_{C1}$ and T$_{C2}$ the behavior is changed to (CAFM-I) with 2D Heisenberg interaction. For T$_{C2}$ < T < T$_{C3}$ the behavior is further changed to higher canting (CAFM-II) with 2D Heisenberg interaction and no interaction between planes of bilayer. Finally above T$_{C3}$, spins become independent and the entire sample behaves as paramagnetic.

The electrical transport analysis shows that there is MIT (Fig. 4) around T$_{C1}$. MIT mechanism can be explained as follows: as the temperature increases, the possibility of lattice polaron formation increases. The lattice polarons start forming at T$_{C1}$ and above T$_{C1}$. Huge number of polarons are formed due to significant lattice distortion, resulting the sample behavior to be insulating^93,96^. The MIT temperature is same as the transition temperature for transition from SR 2D Ising FM to SR ordered competitive FM and AFM canted state having magnetic clusters. This implies that magnetic transition and MIT are intimately related. The conducting region is 2D Ising FM, while the insulating region is an inhomogeneous canted AFM state i.e., long-range and SR magnetic ordering corresponding to long-range and SR charge ordering, respectively. AFM coupling forbid the flow of charge carriers^61,97^. Magnetic transition at T$_{C1}$ is explained by DE interaction. Thus, the polarons are responsible for both magnetic transition and MIT, i.e., above T$_{C1}$ localization of charge carriers increases. As a result, both the resistivity and magnetization decreases with an increase in temperature above T$_{C1}$. The results of critical analysis around T$_{C1}$, T$_{C2}$ and T$_{C3}$ have shown that the behavior of our sample for T < T$_{C1}$, T$_{C1}$ T < T$_{C2}$ and T$_{C2}$ < T < T$_{C3}$ are SR 2D Ising, SR 2D Heisenberg and again SR 2D Heisenberg magnet, which are responsible for existence of non-zero magnetization in these temperature range. The anisotropies observed below these three transition points support the existence of 2D magnetic interaction. Transition at T$_{C2}$ and T$_{C3}$: an ideal 2D Heisenberg magnet is not ordered at finite temperature, however, model allows ordering for finite temperature range with critical fluctuations at sufficiently low temperature^26^. Hence, 2D Heisenberg magnet may have both CAFM and FM coupling with dominating FM coupling, which may cause non-zero magnetic ordering. Infinite size lattice implies ideal 2D Heisenberg magnet that leads to zero ordering at finite temperature (Hohenberg–Mermin–Wagner theorem). Hence, the magnetic lattice of finite size crystal lattice at finite temperature would have non-zero spontaneous magnetization. In our sample the transitions at T$_{C2}$ and T$_{C3}$ are from competing: SR order AFM-I and SR order FM state to SR order AFM-II state and SR order FM state, and SR order AFM-II and SR order FM state to PM state, respectively. Since at these temperatures the observed critical exponent value $\beta$ are 0.27 and 0.254, which correspond to SR 2D Heisenberg model^23–25^, i.e., and this is consistent with small change in magnetization at transitions^47^. A significant deviations in the value of critical exponents at all three transition points have been observed which may be due to the presence of magnetic anisotropy other than exchange interactions such as dipole-dipole interaction, existence of some magnetic random distribution, presence of some magnetic clusters^98–103^. Our sample is showing three transitions having AFM and FM states (see section B), in which spins may be canted due to SE and DE interactions^104^. The canting angle changes by tuning temperature, magnetic field and pressure^47,61^. M-H in Fig. 13, shows no signature of canting at 10 K but at 130 K canting is clearly observed (M-H curve is linear at low field (0−0.25 T) with a neck, then again nearly linear upto 2 T and finally decreases its slope)^61^. Based on neutron diffraction measurements for La$_{1.37}$Sr$_{1.63}$Mn$_{2}$O$_7$, Sonomura et al.^47^ have observed change in magnetic structure from FM to CAFM-I to CAFM-II with increase in temperature. Considering similarities between neutron diffraction data and our observations such as existence of magnetic clusters, we propose that these transitions in our sample correspond to SR 2D-Ising FM to CAFM-I (SR 2D Heisenberg) at T$_{C1}$, CAFM-I to CAFM-II (SR 2D Heisenberg) at T$_{C2}$, and CAFM-II to PM at T$_{C3}$ i.e. a crossover in spin dimensionality from $n = 1$ to $n = 3$. On the basis of the results of neutron scattering for bilayer La$_{1.37}$Sr$_{1.63}$Mn$_{2}$O$_7$^47^, the schematic diagram for canting with temperature corresponding to all the different phases and phase transitions for our sample, is shown in Fig. 13. The canting angle below T$_{C1}$ is negligible due to dominating DE over SE interaction, just above T$_{C1}$ the spins starts canting significantly due to competing DE and SE interaction, and goes on increasing to a maximum value around 90$^{\circ }$ as the temperature is further raised. Inset of Fig. 13 shows the canting angle with temperature at normal pressure for bilayer BL-LSMO-0.315^47^. Now, from the analyses at all the three transition temperatures and the above discussions for canting, the possible schematic phase diagram may be constructed as shown in Fig. 14. Based on the spin structures, the phase diagram consists of three transitions at T$_{C1}$, T$_{C2}$ and T$_{C3}$, and four phases as follows: 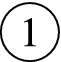: 2D Ising FM in the presence of all the couplings (J$'$, J$_c$ and J$_{ab}$) in which all the spins are almost parallel to the c-axis, 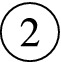: 2D CAFM-I in the presence of J$_c$ and J$_{ab}$ (J$'$ $\sim 0$) in which spins are canted from the c-axis with smaller angle, 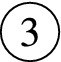: 2D CAFM-II in the presence of only J$_{ab}$ (J$'$ $\sim 0$ and J$_c \sim 0$) in which spins are canted from the c-axis with larger angle, and 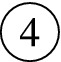: PM (interpreted as $J' \sim 0$, $J_{c} \sim 0$ and $J_{ab} \sim 0$) in which all the spins are randomly oriented.

## Conclusion

Three magnetic transitions have been observed at 101 K, 246 K and 295 K from magnetic measurement in BL-LSMO-0.3. The change in entropy and Arrott analysis have confirmed that these transitions are second order. Critical analysis performed using KF method and MAPs, have yielded that transitions at 101 K, 246 K and 295 K are from SR 2D Ising to SR 2D Heisenberg (CAFM-I), SR 2D Heisenberg (CAFM-I) to another SR 2D Heisenberg (CAFM-II) and SR 2D Heisenberg (CAFM-II) to PM state, respectively. The existence of significant anisotropy at different temperatures below 295 K supports the existence of different magnetic states for 101 K < T < 300 K. The 2D Heisenberg state exhibits canting with AFM interaction as well as FM interaction resulting competing SR FM and AFM clusters. The possible phase diagram corresponding to all four existing magnetic phases has been presented.
